# Case Report: Familial complete androgen insensitivity syndrome across four sisters from childhood to adulthood — a hemizygous AR p.Trp742Leu variant and a 14-year failure to initiate familial cascade evaluation

**DOI:** 10.3389/fendo.2026.1876397

**Published:** 2026-07-01

**Authors:** Burak Çakmak, Savaş Gündoğan, Dilek Tektaş

**Affiliations:** 1 Department of Obstetrics and Gynecology, Mus State Hospital, Mus, Türkiye; 2 Department of Obstetrics and Gynecology, Maslak Acıbadem Hospital, Istanbul, Türkiye; 3 Department of Obstetrics and Gynecology, Şişli Hamidiye Etfal Training and Research Hospital, University of Health Sciences, Istanbul, Türkiye

**Keywords:** 46,XY DSD, AR gene, CAIS, cascade genetic testing, complete androgen insensitivity syndrome, familial case, ligand-binding domain, p.Trp742Leu

## Abstract

We describe a family, with the same biological mother and father, in which complete androgen insensitivity syndrome (CAIS) was identified across 4 phenotypic female sisters spanning a 14-year diagnostic interval from the eldest sister’s diagnosis in adolescence to the proband’s self-presentation as an adult. The proband, a 27-year-old, presented with primary amenorrhea, Tanner 5 breast development, absent axillary and pubic hair, and bilateral inguinal gonads on imaging. Pre-operative hormonal evaluation revealed elevated luteinizing hormone (28.1 mIU/mL), total testosterone within the adult male reference range (8.2 ng/mL; 820 ng/dL; 28.5 nmol/L), and estradiol within the lower female range (42 pg/mL; 154 pmol/L). Sequencing of the *AR* gene in the proband, a 22-year-old affected dizygotic twin sister, and a 14-year-old youngest sister consistently identified the same hemizygous missense variant, NM_000044.6:c.2225G>T, p.(Trp742Leu), in exon 6 within the ligand-binding domain. The variant was absent from gnomAD and classified as pathogenic per American College of Medical Genetics and Genomics/Association for Molecular Pathology (ACMG/AMP) criteria, with co-segregation across three affected siblings. Laparoscopic bilateral gonadectomy was performed in the proband; histopathology showed immature testicular tissue without germ cell neoplasia *in situ*. Two features of this family deserve attention: first, AR Trp742 is well known as a recurrent gain-of-function hot-spot residue in castration-resistant prostate cancer, and its identification as a hemizygous germline variant associated with a complete loss-of-function phenotype underlines the context-dependent interpretation of *AR* variants; second, the 14-year delay between the eldest sister’s diagnosis at age 16 years and the recognition of the family cluster reflects a tractable failure to initiate cascade evaluation rather than a biological challenge. The case argues for embedding pediatric-initiated structured genetic counseling and protocol-driven sibling assessment into the standard care plan for any 46,XY difference of sex development (DSD) diagnosis.

## Introduction

Androgen insensitivity syndrome (AIS) is an X-linked recessive difference of sex development (DSD) caused by hemizygous loss-of-function variants in the androgen receptor (*AR*) gene at Xq11–12 (1, 2). Complete AIS (CAIS), the least virilized form (with a typical female external phenotype), occurs in 46,XY individuals at an estimated worldwide incidence between 1:20,400 and 1:99,100 male live births (3, 4); population-specific estimates for Türkiye are not well established. During fetal life, the sex-determining region of Y (*SRY*) gene drives testicular differentiation, anti-Müllerian hormone secretion by Sertoli cells drives regression of the Müllerian ducts (accounting for the absence of a uterus, cervix, and upper vagina in CAIS), and fetal testosterone production proceeds normally — yet, in CAIS, complete unresponsiveness to androgens prevents Wolffian duct development and external genital virilization, producing a phenotypically female infant with cryptorchid testes (2, 5). At puberty, peripheral aromatization of testosterone drives spontaneous breast development, while primary amenorrhea and minimal or absent pubic and axillary hair complete the characteristic clinical picture (5).

The *AR* gene comprises eight exons, with the N-terminal transactivation domain encoded by exon 1, the DNA-binding domain by exons 2–3, and the ligand-binding domain (LBD) by exons 4–8 (6, 7). More than 1,000 *AR* variants have been catalogued in the Androgen Receptor Gene Mutations Database, with a relative excess of missense changes affecting the LBD (7). Notably, *AR* LBD variants are also recurrent drivers of treatment resistance in advanced and castration-resistant prostate cancer (CRPC), where somatic substitutions at residues such as L702, W742, H875, and T878 confer gain-of-function behavior and convert non-steroidal antiandrogens into partial agonists (8, 9). The same residue may therefore participate in opposing functional phenotypes depending on cellular context, germline versus somatic origin, and the available ligand and co-activator environment.

Although familial clustering is biologically expected in an X-linked recessive condition, most published CAIS reports describe single index cases. Reports analyzing *why* first-degree relatives at risk remain undiagnosed for years after a sentinel familial diagnosis are scarce, even though the structural barriers to cascade testing are well described in other inherited conditions (10). In DSDs specifically, additional layers of complexity (parental wishes regarding the timeline of disclosure, gender-related stigma, and the difficulty of maintaining continuity of care across international borders) may further delay sibling evaluation (11).

We present a family in which CAIS was identified in 4 sisters, with diagnoses spanning childhood, adolescence, and adulthood. Two aspects make this case noteworthy. First, the affected sisters share a hemizygous *AR* variant, p.(Trp742Leu), targeting a residue extensively characterized as a somatic gain-of-function hot-spot in CRPC but, on PubMed-indexed search at the time of writing, sparsely represented as a germline cause of CAIS. Second, although 3 of the 4 sisters were ultimately diagnosed in rapid succession once the proband self-presented to our center, this occurred approximately 14 years after the eldest sister’s diagnosis in adolescence — illustrating not a failure of testing itself, but a failure to initiate cascade evaluation of at-risk relatives despite an established familial diagnosis.

## Case description

A 27-year-old phenotypic female (the proband, Sister 2 in the four-sister birth order) presented to the gynecology outpatient clinic of our tertiary center with primary amenorrhea. She had no significant medical or surgical history. She had been aware that an elder sister had undergone surgery in adolescence “for a similar problem” but had not previously sought formal evaluation. Physical examination revealed Tanner 5 breast development, Tanner 1 pubic hair, and complete absence of axillary hair (12). Body mass index was within the normal range. There were no inguinal masses palpable. Gynecological examination demonstrated typical female external genitalia and a blind-ending vagina approximately 5 cm in length.

Pre-operative hormonal evaluation, performed on the day of initial assessment before any surgical or hormonal intervention, demonstrated a profile consistent with complete androgen resistance (**Table 1**): follicle-stimulating hormone (FSH) 18.2 mIU/mL, luteinizing hormone (LH) 28.1 mIU/mL, total testosterone 8.2 ng/mL (820 ng/dL; 28.5 nmol/L; within the adult male reference range and substantially above the female range), and estradiol 42 pg/mL (154 pmol/L; lower female range). Adrenal androgens, prolactin, and thyroid function were normal. Transabdominal ultrasonography and pelvic magnetic resonance imaging confirmed absence of a uterus, cervix, and ovaries; bilateral solid structures consistent with gonads were located in the inguinal canals (21 × 12 mm left, 29 × 13 mm right).

**Table 1:** Clinical, biochemical, imaging, and molecular characteristics of the 4 affected sisters, with timeline of CAIS diagnosis spanning approximately 14 years.

Parameter	Sister 1 (eldest)	Sister 2 (proband)	Sister 3 (affected twin)	Sister 4 (youngest)
Demographic and timeline				
Age at last assessment (years)	29	27	22	14
Age at first CAIS diagnosis	16 years (in another country)	27 (current presentation)	22 (current presentation)	Karyotype 2019 (7 yrs); AR sequencing 2020 (8 yrs)
Clinical				
Primary amenorrhea	Yes (pre-gonadectomy)	Yes	Yes	Mid-puberty (Tanner 3); menarche not yet reached
Breast development (Tanner)	Tanner 5 (pre-gonadectomy)	Tanner 5	Tanner 5	Tanner 3
Pubic hair (Tanner)	Tanner 1	Tanner 1	Tanner 1	Tanner 1
Axillary hair	Absent	Absent	Absent	Absent
Vaginal length (blind-ending)	Not re-measured at our center	5 cm	4 cm	Not assessed (panel deferred)
Gonad location	Bilateral inguinal (excised)	Bilateral inguinal	Bilateral inguinal	Bilateral inguinal
Uterus/ovaries on imaging	Reported absent (historical)	Absent	Absent	Absent
Hormonal profile (timing as indicated)²				
FSH, mIU/mL (F: 3.5–12.5; M: 1.5–12.4)	Not available	18.2 (pre-op)	13.2 (pre-op)	Not assessed (panel deferred)
LH, mIU/mL (F: 2.4–12.6; M: 1.7–8.6)	Not available	28.1 (pre-op)	22.6 (pre-op)	Not assessed (panel deferred)
Estradiol, pg/mL (F follicular: 30–120; M: 10–40)	65 (239 pmol/L; on long-term oral estrogen)	42 (154 pmol/L; pre-op)	48 (176 pmol/L; pre-op)	Not assessed (panel deferred)
Total testosterone, ng/mL (F: 0.1–0.85; M: 2.8–11.0)	0.6 (60 ng/dL; 2.1 nmol/L; post-gonadectomy, on HRT)	8.2 (820 ng/dL; 28.5 nmol/L; pre-op, before any intervention)	6.5 (650 ng/dL; 22.6 nmol/L; pre-op)	Not assessed (panel deferred)
Cytogenetic				
Karyotype	46,XY (family-reported, prior evaluation)	46,XY (confirmed)	Not performed within scope¹	46,XY (confirmed, 2019)
Molecular (AR sequencing, NM_000044.6)				
*AR variant*	Not tested within scope of this report	**c.2225G>T, p.(Trp742Leu) — confirmed**	**c.2225G>T, p.(Trp742Leu) — confirmed**	**c.2225G>T, p.(Trp742Leu) — confirmed**
Zygosity	—	Hemizygous	Hemizygous	Hemizygous
Management				
Gonadectomy status	Bilateral, performed at age 16 years in another country	Bilateral laparoscopic, current admission	Awaiting elective laparoscopic gonadectomy	Deferred until completion of spontaneous puberty
Histopathology	Not available to us	Immature testicular tissue, Sertoli cells, no GCNIS	Pending	Not applicable
Hormone replacement	Oral estradiol valerate 2 mg/day (continued)	Transdermal 17β-estradiol 50 µg/24 h, twice weekly (post-op)	Planned post-operatively	Not applicable until post-gonadectomy

^¹^ Karyotyping in Sister 3 was not undertaken within the scope of the present report; the diagnosis rests on consistent clinical, biochemical, imaging, and molecular findings.

^2^ Reference ranges shown for both adult female (F) and adult male (M) intervals to allow visual comparison. Hormonal timing for each sister is annotated explicitly: values for the proband and Sister 3 were obtained pre-operatively before any intervention; Sister 1’s values reflect long-term post-gonadectomy estrogen replacement; Sister 4 was in mid-puberty (Tanner 3) at last assessment, with the formal hormonal panel deferred pending completion of puberty. The ages and values of the adult sisters correspond to the 2024 index assessment and those of Sister 4 to the 2026 follow-up. SI conversions: testosterone ng/mL × 3.47 = nmol/L (× 100 = ng/dL); estradiol pg/mL × 3.67 = pmol/L. The specific hormone immunoassay platform used by the referring laboratory could not be verified.

^3^ Country of diagnosis: Sister 1 was diagnosed and underwent gonadectomy in another country; Sisters 2, 3, and 4 were diagnosed and managed at our tertiary center in Türkiye.

AR, androgen receptor; F, adult female reference range; FSH, follicle-stimulating hormone; GCNIS, germ cell neoplasia in situ; HRT, hormone replacement therapy; LH, luteinizing hormone; M, adult male reference range. The bold values indicate the molecularly confirmed AR variant (c.2225G>T, p.(Trp742Leu)) identified by sequencing in the three tested sisters (the proband, Sister 3, and Sister 4).

### Family history and timeline of events

**Figure 1:**
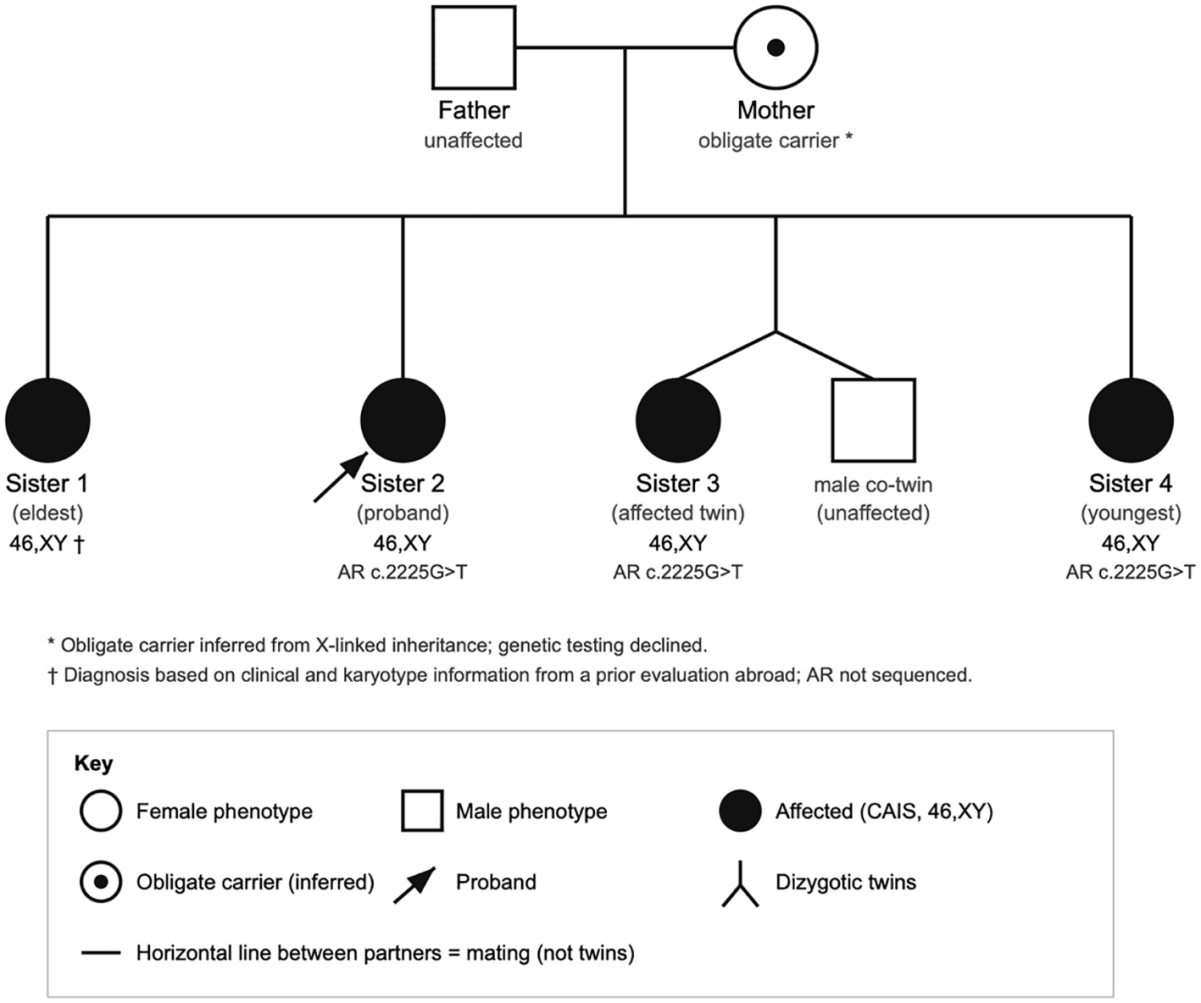
Three-generation pedigree of the family. Circles denote phenotypic females and squares phenotypic males; filled symbols indicate individuals affected by CAIS (46,XY). The mother (circle with a central dot) is an obligate heterozygous carrier by inference from the X-linked inheritance pattern; the father is unaffected. Sister 3 and her dizygotic male co-twin descend by separate lines from a single sibship point without a horizontal twin bar, denoting dizygosity; the co-twin is clinically unaffected. The proband (Sister 2) is indicated by an arrow. Sisters 2, 3, and 4 carry the hemizygous AR c.2225G>T (p.Trp742Leu) variant; Sister 1’s CAIS status rests on family-reported clinical and karyotype information from a prior evaluation in another country and was not confirmed by AR sequencing. Individual ages are not shown on the pedigree and are reported in **Table 1**.

A detailed three-generation pedigree was constructed (**Figure 1**). The family comprised 4 sisters and one clinically unaffected dizygotic male twin (the male co-twin of Sister 3). The parents are nonconsanguineous; the mother declined genetic testing but is an obligate carrier by inference from the X-linked inheritance pattern, and no further family history was available on the maternal side. The clinical timeline of the 4 affected sisters spans approximately 14 years and is best understood as a narrative that begins with the eldest sibling and culminates in the index presentation of the proband and Sister 3.

Sister 1 (29 years old at the family’s index assessment in 2024) was diagnosed with CAIS at age 16 years in another country in late 2010, where she underwent bilateral gonadectomy in 2011 and was started on long-term oral estradiol valerate 2 mg daily. The original cytogenetic report was not transmitted to the family’s subsequent treating clinicians; *AR* sequencing was not performed in this sister within the present scope. Sister 4 (14 years old at most recent follow-up in 2026) was the first family member in whom a molecular diagnosis was pursued: karyotyping performed in 2019 (at age 7 years) confirmed 46,XY, and *AR* sequencing performed in 2020 (at age 8 years) identified the hemizygous c.2225G>T (p.Trp742Leu) variant. Despite this molecular diagnosis being available from 2020, no structured cascade evaluation of the at-risk adult sisters was implemented in the family’s prior clinical contacts. Sister 3 (22 years old at presentation, the affected dizygotic twin sister) and Sister 2 (the proband, 27 years old at presentation) had not undergone formal evaluation when the proband self-presented to our center with primary amenorrhea, prompting family-wide assessment for the first time. The combined timeline therefore spans approximately 14 years between Sister 1’s adolescent diagnosis in late 2010 (age 16 years) and the proband’s adult self-presentation in 2024 (age 27 years); notably, once the proband presented, 3 of the 4 sisters’ diagnoses were consolidated in rapid succession through this single index event rather than through prospective sibling evaluation — underscoring that the limiting step was the initiation of cascade evaluation, not its feasibility.

Sister 3 presented in parallel with the proband: primary amenorrhea, Tanner 5 breast development, absent pubic and axillary hair, a 4 cm blind-ending vaginal pouch, and bilateral inguinal gonads on imaging. Her hormonal profile paralleled that of the proband (**Table 1**). *AR* sequencing identified the same hemizygous variant. Her dizygotic male co-twin was clinically and phenotypically unaffected with normal male external genitalia and pubertal development; karyotyping was not performed in the unaffected co-twin given the absence of clinical indication. Sister 4, on most recent assessment, was in mid-puberty (Tanner 3) with absent pubic and axillary hair; bilateral inguinal gonads were palpable. A formal pubertal hormonal panel was deferred pending completion of pubertal development, in line with current management recommendations (13). Gonadectomy is planned after spontaneous puberty to permit endogenous estrogen exposure and accrual of peak bone mineral density. Written informed consent for clinical evaluation, imaging, surgical management, photography, molecular analysis, and inclusion in this report was obtained from each adult sister and from the parent/legal guardian of Sister 4, with age-appropriate assent additionally obtained from Sister 4 herself.

## Diagnostic assessment, therapeutic intervention, and outcomes

### Genetic analysis

Genomic DNA was extracted from peripheral blood leukocytes of three of the four affected sisters (the proband, Sister 3, and Sister 4) at the regional Genetic Diseases Diagnostic Centre. Sequence analysis of the entire coding region and exon–intron boundaries of the *AR* gene (NM_000044.6) was performed by the reporting laboratory. The exon 1 polymorphic CAG and GGC trinucleotide repeat tracts were not resolvable with the methodology used and were not reported, in line with the laboratory’s standard disclosure for *AR* sequencing (14). Variants were reported using Human Genome Variation Society (HGVS) nomenclature. The candidate variant identified in Sister 4 was subsequently sought and confirmed in the proband and Sister 3 using the same testing pipeline.

Pathogenicity classification followed the 2015 ACMG/AMP joint consensus guidelines (15) with refinements from the ClinGen Sequence Variant Interpretation Working Group (16). Population allele frequencies were assessed against the Genome Aggregation Database (gnomAD v4) (17). The hemizygous *AR* variant — c.2225G>T, p.(Trp742Leu) (genomic coordinate chrX:66,937,371; GRCh38) — was classified as pathogenic based on: PM1 (variant in the AR ligand-binding domain, residues 666–920, a well-established functional domain) (6, 7); PM2_supporting (absent from gnomAD v4) (17); PP1 (co-segregation across three affected sisters in the present family) (15); PP2 (missense variant in a gene where missense variants are a common mechanism of disease) (7); and PP3 (concordant in silico predictions across the standard tool set used by the reporting laboratory). The variant lies in exon 6 as numbered in the diagnostic report (corresponding to exon 5 in transcript-based numbering used in earlier *AR* literature, owing to historical differences in exon designation between genomic and mRNA conventions). At the protein level, Trp742 lies within the AR ligand-binding pocket and contributes to helices H4–H5, a region directly implicated in steroid recognition and antagonist–agonist switching in published structural studies (6, 18).

### Surgical management and histopathology

**Figure 2:**
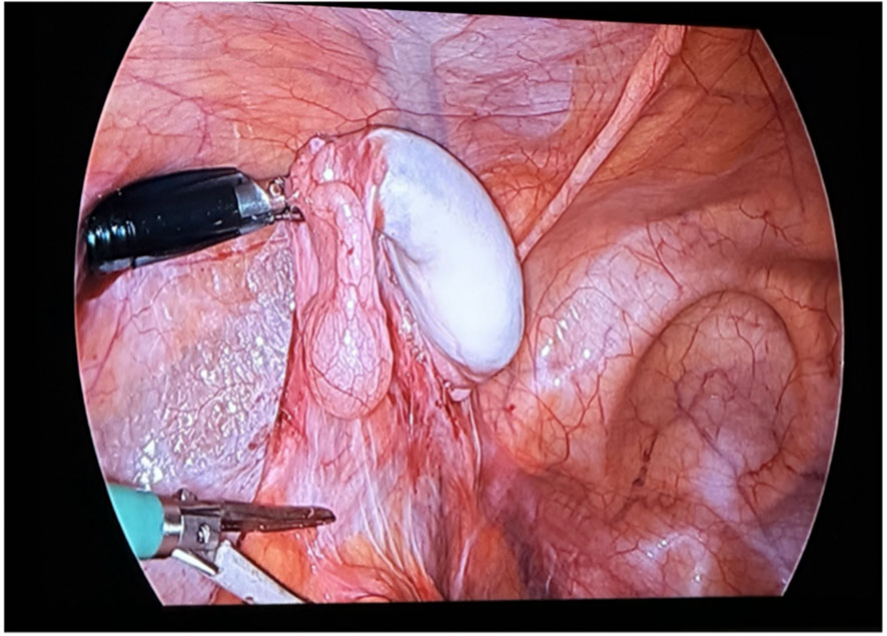
Intra-operative laparoscopic view during bilateral gonadectomy in the proband. The image provides direct anatomical documentation of the in-situ location of the gonad within the inguinal canal and the absence of Müllerian remnants — findings central to the diagnosis that confirm the pre-operative imaging and are difficult to convey by description alone.

**Figure 3:**
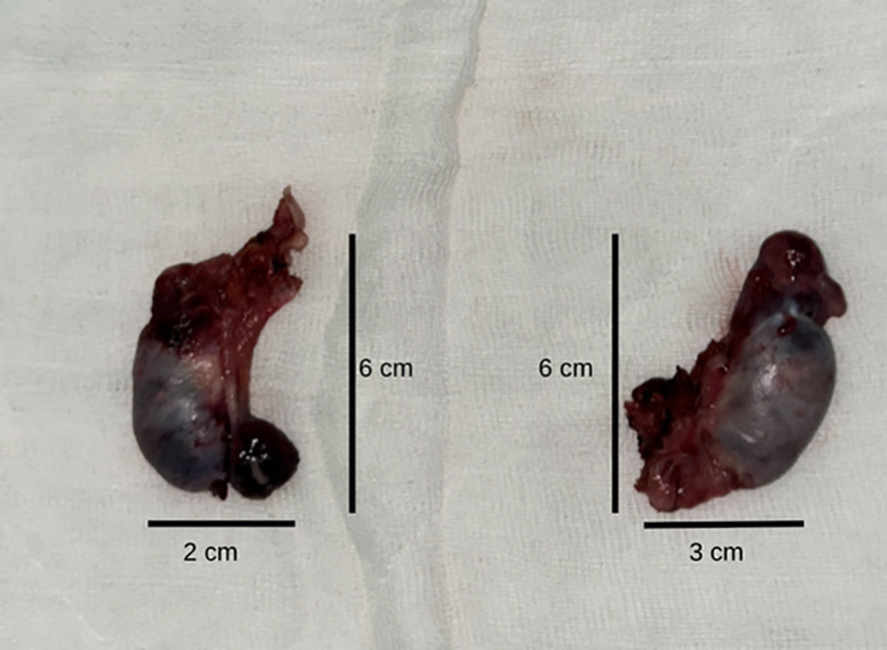
Macroscopic appearance of the excised bilateral gonads. Histopathological examination (reported in the text) demonstrated immature testicular tissue with seminiferous tubules lined by Sertoli cells, associated stromal components, and epididymal structures, and no evidence of germ cell neoplasia in situ (GCNIS) or invasive malignancy.

Following multidisciplinary counseling addressing gonadal tumor risk, fertility implications, and timing of gonadectomy versus surveillance, the proband elected surgical management. Laparoscopic bilateral gonadectomy was performed without complications. Intra-operatively, the gonads lay within the inguinal canals; no Müllerian remnants were observed (**Figure 2**). On macroscopic examination, the two specimens measured 6.0 × 3.0 × 1.5 cm and 6.0 × 2.0 × 1.5 cm (**Figure 3**). Histopathology demonstrated immature testicular tissue with seminiferous tubules lined by Sertoli cells and surrounding stromal components, with associated epididymal structures. Importantly, there was no evidence of germ cell neoplasia *in situ* (GCNIS), pre-malignant lesions, or invasive malignancy in either gonad (19).

### Follow-up and outcomes

Post-operatively, the proband was started on transdermal 17β-estradiol 50 µg/24 h, applied twice weekly, with planned dose titration based on bone mineral density and patient-reported outcomes at six-monthly review during the first two years. Long-term endocrinology and gynecology follow-up was arranged, with review frequency reduced to annual thereafter. Sister 3 is awaiting elective laparoscopic gonadectomy at the time of writing. Sister 1 continues on her established oral estradiol valerate 2 mg/day regimen with no acute complications during the study period. Sister 4 is in active pediatric endocrinology surveillance; her hormonal panel will be performed at completion of puberty.

## Discussion

This four-sister family illustrates two interlocking issues that, taken together, distinguish it from the dominant pattern of single-index CAIS reports: the unusual identification of an *AR* missense variant at a residue otherwise dominated by gain-of-function somatic events in advanced prostate cancer; and a 14-year failure to initiate cascade evaluation that spanned childhood, adolescence, and adulthood despite an established familial diagnosis.

### AR p.Trp742Leu: a context-dependent variant

The AR Trp742 residue lies within helices H4–H5 of the LBD and contributes to the steroid-binding pocket (6, 18). In the somatic literature on castration-resistant prostate cancer (CRPC), Trp742 is one of a small group of recurrent LBD hot-spot residues — together with Leu702, His875, and Thr878 — repeatedly identified through tumour and circulating-cell-free-DNA sequencing (8, 9, 20). In this somatic context, the W742C and W742L substitutions are characterized as gain-of-function changes: they reshape the ligand-binding cavity, allow promiscuous activation by non-canonical ligands, and convert bicalutamide from an antagonist into a partial agonist (20, 21). Functional reporter assays of W742L have shown that, at high dihydrotestosterone concentrations, the mutant supports transcriptional activity exceeding that of the wild-type receptor (20).

The phenotype in our family is the polar opposite — complete loss of androgen action across four affected sisters. Several non-mutually exclusive explanations are plausible. First, gain-of-function behavior in CRPC reflects a tumour-cell context with abundant non-canonical ligands, altered co-activator availability, and amplified or modified *AR* expression — conditions absent during fetal genital development. Second, replacing tryptophan with leucine likely perturbs the structural stability of the ligand-bound conformation *in vivo*, even where *in vitro* reporter assays in heterologous cells suggest residual or paradoxically increased activity. Third, the germline hemizygous state in a 46,XY individual exposes the variant’s effect on physiological androgen signaling without the buffering of a second functional allele. The cytogenetic report documented prior literature reporting of this variant (PMID: 11788645); however, on targeted re-search of current PubMed-indexed primary literature we did not retrieve published reports of p.Trp742Leu as a *germline* cause of CAIS, although the residue is well represented in somatic cancer datasets (7, 9). The methodological lesson is more important than the claim of priority: variant interpretation in DSD must integrate population data, in silico prediction, ACMG/AMP criteria (15, 16), and phenotype-aware reasoning, rather than relying solely on the prior report status of a residue.

### Diagnostic delay and the failure of cascade evaluation

The most clinically actionable feature of this family is the 14-year interval between Sister 1’s diagnosis at age 16 years (with subsequent gonadectomy abroad) and the proband’s self-presentation at age 27 years. During this interval, the dizygotic twin sister and the youngest sister remained without comprehensive evaluation in the family’s clinical contacts, even though the youngest sister’s molecular diagnosis had been available since 2020. This is not a failure of biology — *AR* is X-linked recessive and familial clustering is precisely what is biologically expected — but a failure of clinical infrastructure. Several plausible contributors deserve to be named. First, in many health systems no formal protocol triggers cascade evaluation of first-degree relatives once a 46,XY DSD is diagnosed in an adolescent; cascade testing pathways are well developed for hereditary cancer syndromes (10) but considerably less consistent for DSD. Second, genetic counseling at Sister 1’s initial diagnosis — performed in another country and a different health-system context — does not appear to have generated a written family-screening recommendation. Third, the social and psychosocial particularities of DSD, including parental wishes regarding the timeline of disclosure, gender-related stigma, and a tendency for family-level information to be held by parents rather than communicated to at-risk relatives, can lead families to avoid further evaluation (11, 22). These dynamics are compounded by the ethical complexity of disclosing one sibling’s diagnosis to another: informing the younger sisters would itself have required the eldest sister’s consent to share her own diagnosis, and families may defer disclosure out of a wish to protect children from stigma, uncertainty about how and when to communicate a 46,XY DSD diagnosis, and concern about its psychological impact. Fourth, the family’s relocation across borders interrupted longitudinal care.

Concrete and largely pragmatic interventions could prevent comparable delays. Any 46,XY DSD diagnosis should generate a structured family-screening communication naming the at-risk first-degree relatives and the recommended assessment, with a designated coordinating clinician. Genetic counseling should be a documented, billable component of the management plan rather than a discretionary referral. In adolescent girls presenting with primary amenorrhea, a positive family history of “infertility,” “no menstruation,” or unexplained inguinal hernia in sisters should prompt karyotyping and *AR* analysis at low threshold. Once a familial variant is known, cascade testing of at-risk relatives can rely on targeted single-variant analysis rather than full-gene sequencing, which is substantially less costly and more readily accessible. For families with cross-border health-care trajectories, a portable patient-held summary of the molecular diagnosis and recommended sibling assessment would meaningfully reduce fragmentation. Critically, the entry point for cascade evaluation in DSD is most often the pediatric encounter — implementation responsibility therefore rests substantially with pediatric endocrinologists.

### Management considerations

The four sisters in this family span the heterogeneity of contemporary CAIS management. Sister 1 underwent gonadectomy at age 16, in keeping with the older paradigm prioritizing presumed malignancy risk; the proband elected gonadectomy as an adult; Sister 3 awaits elective surgery; and Sister 4’s surgery is deferred until completion of spontaneous puberty. Contemporary evidence indicates that the lifetime risk of malignant germ cell tumour in the CAIS gonad is far lower than once thought, particularly during childhood and adolescence (well under 1% before puberty), rising with age (13, 19, 23). International expert practice has shifted in favor of deferring gonadectomy until after spontaneous puberty (3, 13, 23, 24). Hormone replacement after gonadectomy is essential for bone health and quality of life (5, 25); oral or transdermal estrogen remains the standard of care (5, 25), with injectable estrogen preparations also in common use in some settings such as the United States; the dose is titrated to clinical response and serum estradiol. The randomized trial by Birnbaum et al. (26) reported preliminary signals favoring testosterone for selected patient-reported outcomes in adult CAIS, but the evidence base remains limited and testosterone is not currently part of routine post-gonadectomy care.

### Strengths and limitations

The principal strength of this report is co-segregation of the *AR* p.(Trp742Leu) variant across three affected sisters, supporting the pathogenicity classification beyond what is achievable from single-case reports. Limitations include: Sister 1 has not undergone *AR* sequencing, with her CAIS status resting on family-reported clinical and karyotype information; karyotyping in Sister 3 was not undertaken within the present scope; no functional assays were performed on patient-derived material; the specific hormone immunoassay platforms used by the referring laboratories could not be verified, so reported values should be interpreted with assay-related caveats rather than against liquid chromatography–tandem mass spectrometry references; and the proposed reasons for diagnostic delay are interpretive rather than systematically measured.

### Take-away lessons

CAIS should be considered in any phenotypic female adolescent with primary amenorrhea, sparse or absent pubic and axillary hair, and a 46,XY karyotype, particularly when the family history includes “infertility,” “absent menstruation,” or unexplained inguinal hernias in sisters. The same *AR* missense change may produce divergent phenotypes in germline versus somatic context. Most importantly, structured cascade evaluation — initiated at the pediatric encounter and carried forward across the lifespan — is a clinically tractable intervention that, while not without resource and counseling costs, could have prevented the kind of 14-year diagnostic gap observed in this family.

### Patient perspective

A formal patient perspective statement was not included in the present report; the proband consented to publication of clinical, genetic, and surgical data but elected not to provide a personal narrative for inclusion. Her decision was respected as part of the informed-consent process.

## Data Availability

The original contributions presented in the study are included in the article/supplementary material. Further inquiries can be directed to the corresponding author.

## References

[1] Hughes IA Davies JD Bunch TI Pasterski V Mastroyannopoulou K MacDougall J . Androgen insensitivity syndrome. Lancet. (2012) 380:1419–28. doi: 10.1016/S0140-6736(12)60071-3 22698698

[2] Tyutyusheva N Mancini I Baroncelli GI D’Elios S Peroni D Meriggiola MC . Complete androgen insensitivity syndrome: from bench to bed. Int J Mol Sci. (2021) 22:1264. doi: 10.3390/ijms22031264 33514065 PMC7865707

[3] Lanciotti L Cofini M Leonardi A Bertozzi M Penta L Esposito S . Different clinical presentations and management in complete androgen insensitivity syndrome (CAIS). Int J Environ Res Public Health. (2019) 16:1268. doi: 10.3390/ijerph16071268 30970592 PMC6480640

[4] Wang C Tian Q . Molecular pathogenesis, diagnosis, and management challenges in complete androgen insensitivity syndrome. Front Endocrinol (Lausanne). (2025) 16:1600343. doi: 10.3389/fendo.2025.1600343 41163677 PMC12558735

[5] Kosti K Athanasiadis L Goulis DG . Long-term consequences of androgen insensitivity syndrome. Maturitas. (2019) 127:51–4. doi: 10.1016/j.maturitas.2019.06.004 31351520

[6] Gao W Bohl CE Dalton JT . Chemistry and structural biology of androgen receptor. Chem Rev. (2005) 105:3352–70. doi: 10.1021/cr020456u 16159155 PMC2096617

[7] Gottlieb B Beitel LK Nadarajah A Paliouras M Trifiro M . The androgen receptor gene mutations database: 2012 update. Hum Mutat. (2012) 33:887–94. doi: 10.1002/humu.22046 22334387

[8] Watson PA Arora VK Sawyers CL . Emerging mechanisms of resistance to androgen receptor inhibitors in prostate cancer. Nat Rev Cancer. (2015) 15:701–11. doi: 10.1038/nrc4016 26563462 PMC4771416

[9] Robinson D Van Allen EM Wu YM Schultz N Lonigro RJ Mosquera JM . Integrative clinical genomics of advanced prostate cancer. Cell. (2015) 161:1215–28. doi: 10.1016/j.cell.2015.05.001 26000489 PMC4484602

[10] Roberts MC Foss K Henderson GE Powell SN Saylor KW Weck KE . Exploring family communication preferences in hereditary breast and ovarian cancer and Lynch syndrome: a national Canadian survey. J Community Genet. (2024) 15:485–97. doi: 10.1007/s12687-024-00720-z 39046652 PMC11410744

[11] Weidler EM Baratz A Muscarella M Hernandez SJ van Leeuwen K . A shared decision-making tool for individuals living with complete androgen insensitivity syndrome. Semin Pediatr Surg. (2019) 28:150841. doi: 10.1016/j.sempedsurg.2019.150841 31668289 PMC7208826

[12] Marshall WA Tanner JM . Variations in pattern of pubertal changes in girls. Arch Dis Child. (1969) 44:291–303. doi: 10.1136/adc.44.235.291 5785179 PMC2020314

[13] Cools M Looijenga L . Update on the pathophysiology and risk factors for the development of Malignant testicular germ cell tumors in complete androgen insensitivity syndrome. Sex Dev. (2017) 11:175–81. doi: 10.1159/000477921 28719895

[14] Wieacker PF Knoke I Jakubiczka S . Clinical and molecular aspects of androgen receptor defects. Exp Clin Endocrinol Diabetes. (1998) 106:446–53. doi: 10.1055/s-0029-1212014 10079022

[15] Richards S Aziz N Bale S Bick D Das S Gastier-Foster J . Standards and guidelines for the interpretation of sequence variants: a joint consensus recommendation of the American College of Medical Genetics and Genomics and the Association for Molecular Pathology. Genet Med. (2015) 17:405–24. doi: 10.1038/gim.2015.30 25741868 PMC4544753

[16] Abou Tayoun AN Pesaran T DiStefano MT Oza A Rehm HL Biesecker LG . Recommendations for interpreting the loss of function PVS1 ACMG/AMP variant criterion. Hum Mutat. (2018) 39:1517–24. doi: 10.1002/humu.23626 30192042 PMC6185798

[17] Karczewski KJ Francioli LC Tiao G Cummings BB Alföldi J Wang Q . The mutational constraint spectrum quantified from variation in 141,456 humans. Nature. (2020) 581:434–43. doi: 10.1038/s41586-020-2308-7 32461654 PMC7334197

[18] Pereira de Jésus-Tran K Côté PL Cantin L Blanchet J Labrie F Breton R . Comparison of crystal structures of human androgen receptor ligand-binding domain complexed with various agonists reveals molecular determinants responsible for binding affinity. Protein Sci. (2006) 15:987–99. doi: 10.1110/ps.051905906 16641486 PMC2242507

[19] Cools M Drop SLS Wolffenbuttel KP Oosterhuis JW Looijenga LHJ . Germ cell tumors in the intersex gonad: old paths, new directions, moving frontiers. Endocr Rev. (2006) 27:468–84. doi: 10.1210/er.2006-0005 16735607

[20] Lallous N Volik SV Awrey S Leblanc E Tse R Murillo J . Functional analysis of androgen receptor mutations that confer anti-androgen resistance identified in circulating cell-free DNA from prostate cancer patients. Genome Biol. (2016) 17:10. doi: 10.1186/s13059-015-0864-1 26813233 PMC4729137

[21] Hara T Miyazaki J Araki H Yamaoka M Kanzaki N Kusaka M . Novel mutations of androgen receptor: a possible mechanism of bicalutamide withdrawal syndrome. Cancer Res. (2003) 63:149–53 12517791

[22] Sandberg DE Mazur T . A noncategorical approach to the psychosocial care of persons with DSD and their families. In: Kreukels BPC Steensma TD de Vries ALC , editors. Gender Dysphoria and Disorders of Sex Development. Springer, Boston (2014). p. 93–114.

[23] Tack LJW Maris E Looijenga LHJ Hannema SE Audi L Köhler B . Management of gonads in adults with androgen insensitivity: an international survey. Horm Res Paediatr. (2018) 90:236–46. doi: 10.1159/000493645 30336477

[24] Deans R Creighton SM Liao LM Conway GS . Timing of gonadectomy in adult women with complete androgen insensitivity syndrome (CAIS): patient preferences and clinical evidence. Clin Endocrinol (Oxf). (2012) 76:894–8. doi: 10.1111/j.1365-2265.2012.04330.x 22211628

[25] Ko JKY King TFJ Williams L Creighton SM Conway GS . Hormone replacement treatment choices in complete androgen insensitivity syndrome. Endocr Connect. (2017) 6:375–9. doi: 10.1530/EC-17-0083 28615185 PMC5527352

[26] Birnbaum W Marshall L Werner R Kulle A Holterhus PM Rall K . Oestrogen versus androgen in hormone-replacement therapy for complete androgen insensitivity syndrome: a double-blind, randomised, placebo-controlled, crossover trial. Lancet Diabetes Endocrinol. (2018) 6:771–80. doi: 10.1016/S2213-8587(18)30197-9 30075954

